# Propofol Treatment Inhibits Constitutive Apoptosis in Human Primary Neutrophils and Granulocyte-Differentiated Human HL60 Cells

**DOI:** 10.1371/journal.pone.0129693

**Published:** 2015-06-10

**Authors:** Chung-Hsi Hsing, Chia-Ling Chen, Wei-Chieh Lin, Chiou-Feng Lin

**Affiliations:** 1 Department of Anesthesiology, Chi Mei Medical Center, Tainan, Taiwan; 2 Department of Anesthesiology, College of Medicine, Taipei Medical University, Taipei, Taiwan; 3 Translational Research Center, Taipei Medical University, Taipei, Taiwan; 4 Department of Internal Medicine, College of Medicine, National Cheng Kung University, Tainan, Taiwan; 5 Graduate Institute of Medical Sciences, College of Medicine, Taipei Medical University, Taipei, Taiwan; 6 Department of Microbiology and Immunology, College of Medicine, Taipei Medical University, Taipei, Taiwan; Singapore Immunology Network, SINGAPORE

## Abstract

Apoptosis regulation is essential for neutrophil homeostasis. We previously demonstrated that a process involving glycogen synthase kinase (GSK)-3β determines neutrophil apoptosis. As for this apoptotic process, an overdose of propofol (2,6-Diisopropylphenol; 25 μg/ml or 140 μM) also causes GSK-3β-mediated macrophage apoptosis; however, the early deactivation of GSK-3β with low-dose propofol has been shown. Therefore, we hypothesize that low-dose propofol may induce neutrophil survival via GSK-3β inactivation. Following *in vitro* culture, the therapeutic concentration of propofol (10 μg/ml or 56 μM) treatment decreased constitutive apoptosis in isolated human primary neutrophils and in granulocyte-differentiated HL60 cells after all-*trans* retinoic acid (1 μM) treatment. The inactivation of phosphatidylinositol 3-kinase (PI3-kinase)/AKT and the activation of GSK-3β results in myeloid cell leukemia 1 (Mcl-1) down-regulation, the loss of the mitochondrial transmembrane potential, and caspase-3 activation in these cells, which is accompanied by apoptosis. Notably, propofol treatment attenuates these effects in a PI3-kinase-regulated manner. We found that propofol initiates PI3-kinase/AKT-mediated GSK-3β inactivation and Mcl-1 stabilization, rescuing the constitutive apoptosis in primary neutrophils and granulocyte-differentiated acute promyelocytic leukemia HL60 cells.

## Introduction

Neutrophils, also called polymorphonuclear leukocytes (PMNs), are the major population of circulating leukocytes that participate in inflammation as the first line of defense against invading pathogens, exerting their effects through phagocytosis. To control the homeostasis of neutrophils, these cells have the shortest lifespan, limiting the dysregulation of inflammatory activation [1,2]. Without or with phagocytosis, constitutive or spontaneous apoptosis may control the half-life of neutrophils. During inflammation and drug treatment, a prolong survival response can be found in neutrophils and its molecular mechanisms underlying cell survival are under investigation [1,2].

The anesthetic propofol (2,6-di(propan-2-yl)phenol), a short-acting and widely used, intravenously administered hypnotic/amnestic agent, has immunomodulating actions by modulating the production of pro-inflammatory mediators and responses in activated neutrophils [3]. However, propofol treatment abuse causes severe complications in patients with critical illnesses, which is called propofol infusion syndrome and is accompanied by the loss of peripheral leukocytes [4]. For clinical medication, a safe range for the anesthetic concentration of propofol is less than 5 mg/kg/h, which provides satisfactory sedation [5]. A significant increase in the neutrophil count has been reported in patients with propofol sedation during gynecologic laparoscopy [6]. It is worth investigating the effects of propofol not only on the inflammatory activation but also on the cell fate of neutrophils.

Regarding overexpressing glycogen synthase kinase (GSK)-3β, a serine/threonine protein kinase, causes cell apoptosis, the pro-apoptotic role of glycogen synthase kinase (GSK)-3β, which is negatively regulated by phosphatidylinositol 3-kinase (PI3-kinase)/AKT, has been widely investigated [7,8]. GSK-3β phosphorylates pro-apoptotic B-cell lymphoma 2 (Bcl-2)-associated protein X (Bax) at serine 163, resulting in activation and mitochondrial translocation, which in turn forms pores in the mitochondrial membrane [9]. The translocation of Bax into the mitochondria disrupts the mitochondrial transmembrane potential (MTP), which is followed by the release of cytochrome *c* and the induction of apoptosome formation followed by caspase-9 and -3 cascade activation. GSK-3β also phosphorylates anti-apoptotic myeloid cell leukemia 1 (Mcl-1), an anti-apoptotic member of the Bcl-2 family of apoptosis-regulating proteins, at serine 159, thus inactivating Mcl-1 and promoting mitochondrial injury [10]. Mcl-1 blocks the loss of MTP by binding and sequestering the pro-apoptotic proteins Bcl-2 homologous antagonist killer, Bak, and Bax. The phosphorylation of Mcl-1 by GSK-3β results in its degradation via an ubiquitin-proteasome system. We currently showed that GSK-3β inhibition decreases constitutive neutrophil apoptosis in primary neutrophils and in granulocyte-differentiated cells [11,12].

To control the lifespan of neutrophils, after they differentiate from bone marrow, neutrophils die via constitutive apoptosis [1,2]. The involvement of death receptors, kinases, Bcl-2 family proteins, reactive oxygen species (ROS), the proteasome, proteases, and caspases is required to control the apoptotic pathway associated with constitutive neutrophil death [13,14]. During sepsis, the reduced number of circulating neutrophils, called neutropenia, is a marker of the disease severity [15,16]. It is worth noting that propofol confers cytoprotection against multiple organ septic dysfunction and failure. Though an anti-inflammatory response following propofol treatment has been proposed [3], it is unknown whether propofol modulates the life-span of neutrophils as part of the host defense against microbial infection. In this study, we investigated the cytoprotective effects of propofol treatment against constitutive apoptosis in primary neutrophils and granulocyte-differentiated acute promyelocytic leukemia (APL) HL60 cells. The potential effects of propofol treatment on the PI3-kinase/AKT/GSK-3β/Mcl-1 signaling pathway axis were also studied.

## Materials and Methods

### Cells, culture condition, and reagents

We included healthy volunteer (n = 10) as the control subjects. The protocols and procedures were approved by the Institutional Review Board of National Cheng Kung University Hospital with written informed consent obtained from healthy volunteers. Human peripheral whole blood was suspended in 4% dextran (Sigma-Aldrich) at room-temperature for 30 min and collected supernatant. Then, human peripheral blood leukocyte (PBL) suspension was gently overlaid onto Ficoll-paque plus (GE Healthcare, Amersham Biosciences, Sweden), and centrifuged at 1,800 rpm for 20 min. Pellet containing PMNs were collected, washed, and resuspended in RPMI 1640 medium (Invitrogen Life Technologies, Rockville, MD) with 10% fetal bovine serum (FBS, Invitrogen Life Technologies). Human APL HL60 cells (ATCC CCL-240) were kindly provided by Dr. Chi-Chang Shieh, Institute of Clinical Medicine, National Cheng Kung University, Taiwan. The cells were cultured in RPMI-1640 medium (Invitrogen Life Technologies, Carlsbad, CA, USA) supplemented with 10% heat-inactivated FBS and maintained at 37°C with 5% CO_2_. All of the cell culture media and reagents were obtained from Invitrogen Life Technologies (Carlsbad, CA, USA). Propofol (2,6-Diisopropylphenol) and all-*trans* retinoic acid (ATRA) were purchased from Sigma-Aldrich (St. Louis, MO, USA) and dissolved in dimethyl sulfoxide (DMSO) for stock. Under experiments, stock propofol was diluted in medium as the indicated concentrations. The PI3-kinase inhibitor LY294002 was obtained from Cayman (Ann Arbor, MI, USA) and dissolved in DMSO.

### Liu’s staining

The morphological assessment of differentiated cells was performed using Liu’s staining method, a modified method of Romanowsky staining. Cells (5 × 10^4^) were fixed on slides using a cytocentrifuge (Cytospin 4, Thermo Scientific, Runcorn, Cheshire, UK), and the samples were processed with Liu’s stain (TONYAR Biotech, Taoyuan, Taiwan). First, we added 0.5 ml LiuA reagent containing Eosin Y (for cytoplasm staining) to the slides for 40 seconds at room temperature and then added 1 ml LiuB reagent containing Azur I and methylene azure (for nuclei stain) for 2 min at room temperature, which was followed by gentle mixing of the reagents by blowing on the slides. The slides were washed with running water for 2 min and then air-dried. The morphology of the cells was examined under a light microscope. A condensed chromatin staining can be clarified as an apoptotic characteristic according to the results of Liu’s staining.

### Apoptosis assay

Cells were resuspended and fixed by adding 1 ml of ice-cold 70% ethanol to phosphate-buffered saline (PBS) and were then stored at 4°C. Before the analysis, the fixed cells were washed in PBS and incubated with propidium iodide (PI) staining solution [(0.04 mg/ml of PI (Sigma-Aldrich) and 0.1 mg/ml of RNase A (Sigma-Aldrich)] for 30 min at room temperature. The cells were analyzed using a flow cytometer (FACSCalibur; BD Biosciences, San Jose, CA, USA) with an excitation at 488 nm and emission in the FL-2 channel (565–610 nm). We analyzed ten thousand cells per sample. Small cellular debris was excluded by gating on a forward scatter plot, and the apoptotic cells were gated and quantified in the sub-G_1_ phase. Annexin V/PI staining was performed according to the manufacturer's instructions (eBioscience, San Diego, CA, USA). The cells were detected in the FL-1 (480–530 nm) and FL-2 channels (565–610 nm) using the FACS Calibur.

### Nitroblue tetrazolium (NBT) assay

HL60 cells (1 × 10^5^ cells/ml) were seeding in a 96-well plate and treated with ATRA. A 10-μl aliquot of NBT solution, composed of 10 mg/ml NBT (Sigma-Aldrich) and 2 μg/ml PMA, was added to each well, and then cells were incubated for 30 min at 37°C. After removing the supernatant, the purple crystals were dissolved in DMSO. The absorbance was detected using a microplate reader (SpectraMax 340PC; Molecular Devices, Sunnyvale, CA, USA).

### WST-8 assay

To determine cell viability, a Cell Counting Kit 8 (WST-8) (Dojindo Molecular Technologies, Kumamoto, Japan) was used according to the manufacturer’s instructions. A microplate reader (SpectraMax 340PC) was used to measure the absorbance at 450 nm.

### Lactate dehydrogenase (LDH) assay

An extracellular LDH assay (Cytotoxicity Detection Kit; Roche Applied Science, Mannheim, Germany) was performed to determine cell death; LDH activity was assayed using a colorimetric assay according to the manufacturer’s instructions. A microplate reader (Spectra MAX 340PC) was used to measure the absorbance at 490 nm, and the data were analyzed with Softmax Pro software.

### Western blot analysis

Forty micrograms of protein from each sample was separated by SDS-PAGE and then transferred to a polyvinylidene difluoride membrane (Millipore, Billerica, MA, USA). After blocking, the blots were developed with a series of antibodies, as indicated. Rabbit antibodies specific for human AKT and phosphorylated AKT (Ser^473^), GSK-3β and phosphorylated GSK-3β (Ser^9^), Mcl-1 (Cell Signaling Technology, Beverly, MA, USA), and phosphorylated GSK-3β (Tyr^216^) (Abcam, Cambridge, UK) and β-actin (Sigma-Aldrich) were used. The blots were incubated with horseradish peroxidase-conjugated anti-goat, rabbit or mouse IgG (Cell Signaling Technology) and developed using an ECL development kit (Millipore). The relative signal intensity was quantified using the ImageJ software (version 1.41o) from W. Rasband (National Institutes of Health, Bethesda, MD, USA). The quantitative results of optimal band density were used for labeling the changes by the ratio of phosphorylated protein compared with the total protein and ratio of protein compared to β-actin.

### PI3-kinase activity assay

We used a commercial a PIP3 Mass ELISA kit (K-2500s, Echelon Biosciences, Salt Lake City, UT) to detect the activity of PI3-kinase according to the manufacturers’ instructions.

### Mitochondrial functional assay

The loss of MTP value was determined using rhodamine 123 (Sigma-Aldrich). Cells were incubated with 50 μM rhodamine 123 in cultured medium for 30 min at 37°C. After they were washed with PBS, the cells were resuspended in cold PBS and immediately underwent flow cytometric analysis (FACSCalibur) with an excitation wavelength of 488 nm and emission detected with the FL-1 channel (515–545 nm). The levels of MTP loss were reported as a percentage (%) of the total cells.

### Caspase-3 activity assay

Cellular caspase-3 activation was determined using the ApoAlert caspase colorimetric assay kits (Clontech, Palo Alto, CA) for caspase-3 according to the manufacturer’s instructions. Optical density (OD) measurements were made using a microplate reader (Molecular Devices).

### Statistical analysis

The values are expressed as the means ± standard deviation (SD). The groups were compared using Student’s two-tailed unpaired *t-*test or a one-way analysis of variance analysis. A *P*-value of 0.05 was considered significant.

## Results

### Propofol treatment inhibits constitutive apoptosis in isolated primary neutrophils

**Fig 1:**
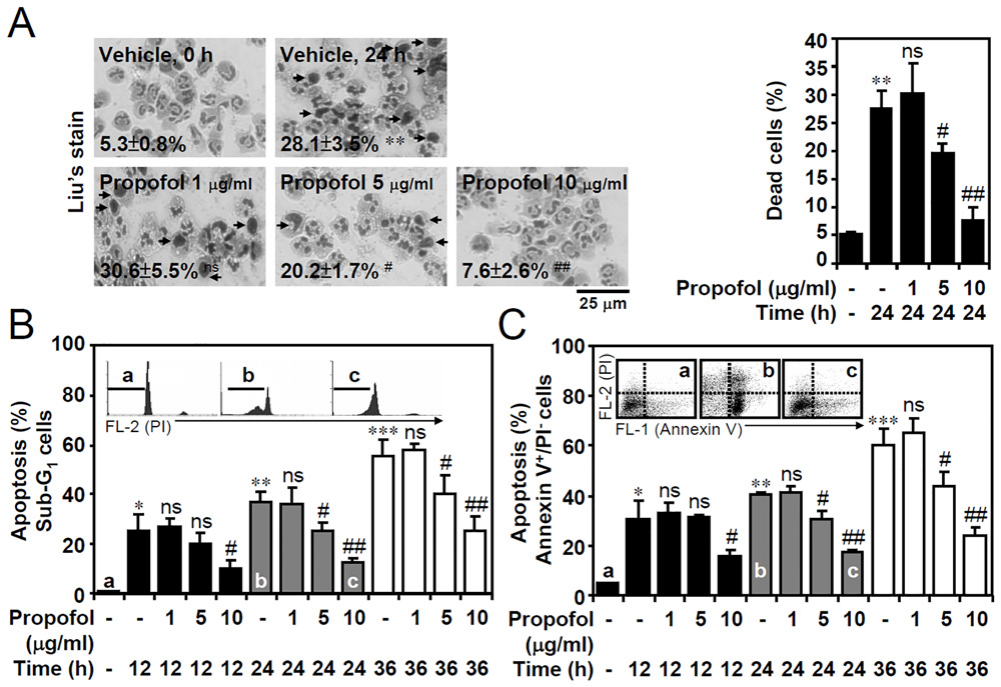
Propofol treatment prevents constitutive apoptosis of human primary neutrophils under *in vitro* culture conditions. (A) Isolated human primary neutrophils were treated with DMSO (the same volume of dilution used in preparation of 10 μg/ml of propofol) or propofol (1, 5, or 10 μg/ml) in 24 h of *in vitro* culture. DMSO was used as the vehicle control. A representative Liu’s stain and the percentages of cells (mean ± SD of three experiments) show apoptotic neutrophils characterized by DNA condensation (dead cell as arrowed). (B) PI and (C) Annexin V-FITC/PI staining followed by flow cytometric analysis determined the levels of apoptosis (sub-G_1_) and early apoptosis (Annexin V^+^/PI^-^) in neutrophils treated with or without propofol for the indicated time points. The original histograms and dot-plots of staining are also shown for a, b, and c groups as indicated. For all experiments, the percentages of apoptotic cells are shown as the means ± SD of three individual experiments. *P < 0.05, **P < 0.01, and ***P < 0.001 compared with untreated at 0 h. #*P <* 0.05 and ##*P <* 0.01 compared with untreated at each time points. ns, not significant compared with untreated at each time points.

We hypothesized that a pro-survival role of propofol prevents constitutive apoptosis in neutrophils. Isolated human primary PMN cells were purified from the PBL of whole blood [11] and the purity was confirmed by CD177 staining (data not shown). By revealing the presence of chromatin condensation, Liu’s stain showed that propofol significantly inhibited constitutive apoptosis in isolated human primary neutrophils following 24 h of *in vitro* culture (Fig 1A). Nuclear PI staining followed by flow cytometric analysis showed that the neutrophils underwent apoptosis (as shown in the sub-G_1_ phase), whereas propofol significantly prevented the apoptotic effects (Fig 1B). Annexin V-FITC/PI staining showed the presence of Annexin V^+^/PI^-^ cells confirmed the effects of propofol on preventing early apoptosis (Fig 1C). These results indicate that propofol sequentially prevents constitutive apoptosis in primary neutrophils.

### Propofol treatment inhibits constitutive apoptosis in granulocyte-differentiated HL60 cells

**Fig 2:**
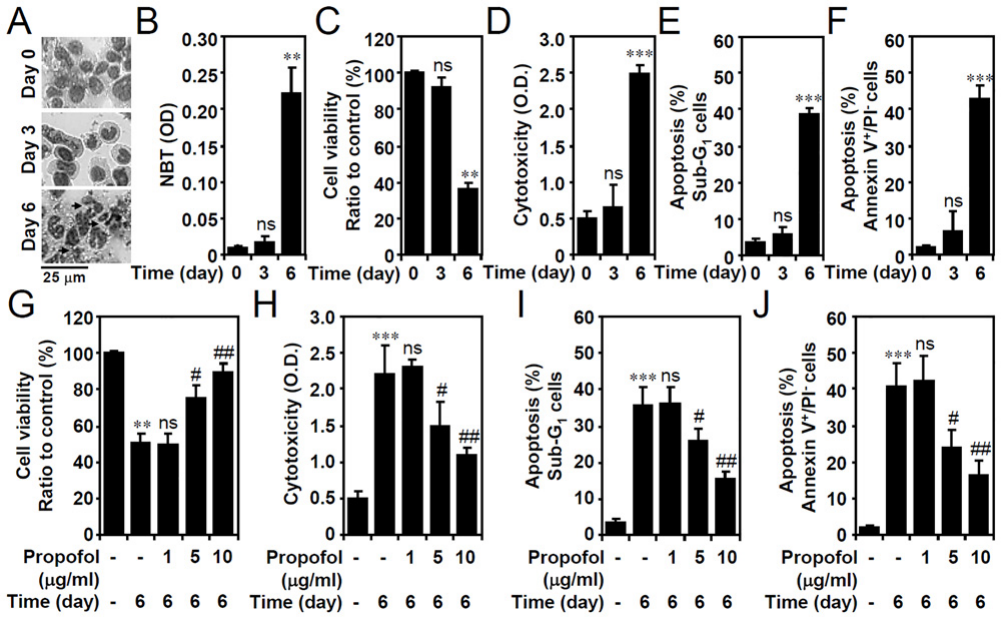
Propofol treatment reduces constitutive apoptosis in ATRA-differentiated granulocytic HL60 cells. HL60 cells were treated with ATRA (1 μM). (A) Representative Liu’s staining showing nuclear morphological changes and cytoplasmic vacuolation (arrowed). (B) A time course of the change in the NBT reduction was performed to assess granulocytic differentiation. OD, optical density. WST-8 activity (compared to the normalized control as 100%) (C and G), LDH release (OD) (D and H), PI (E and I), and PI/Annexin V-FITC (F and J) staining followed by flow cytometric analysis showed alterations in the cell viability, cytotoxicity, and apoptosis (% of apoptotic cells gated on sub-G_1_ and Annexin V^+^/PI^-^), respectively, in ATRA-treated cells without (C-F) or with propofol treatment (G-J) for the indicated time points. The data are shown as the means ± SD of three individual experiments. **P < 0.01 and ***P < 0.001 compared with untreated at 0 h. #*P <* 0.05 and ##*P <* 0.01 compared with untreated at day 6. ns, not significant compared with untreated at 0 h or day 6.

To illustrate a model of granulocytic cell apoptosis other than in primary neutrophils, as demonstrated in our previous study [12], a clinical concentration (1 μM) of ATRA was used to spontaneously direct APL HL60 cells toward granulocytic differentiation, which was followed by apoptosis. ATRA treatment caused nuclear morphological changes in cells from mononuclear to segmented (Fig 2A). ATRA significantly increased the NBT-reducing ability 6 days post-treatment (Fig 2B), indicating that superoxide anions are generated during granulocytic differentiation. In differentiated cells, ATRA not only triggered granulocytic differentiation, hut it also caused a significant growth inhibition (Fig 2C) and cellular cytotoxicity (Fig 2D). A PI-based flow cytometric analysis (Fig 2E) and flow cytometric analysis of annexin V-PI staining (Fig 2F) revealed significant ATRA-induced apoptosis characterized by DNA fragmentation and condensation as well as phosphatidylserine externalization. Notably, all of the results showed that propofol treatment significantly retarded ATRA-induced growth inhibition (Fig 2G), cellular cytotoxicity (Fig 2H), and cell apoptosis (Fig 2I and 2J). These results suggest that the pharmacological treatment of differentiated cells with propofol significantly abolished ATRA-induced apoptosis.

### Propofol treatment reverses AKT inactivation and GSK-3β activation in a PI3-kinase-regulated manner

**Fig 3:**
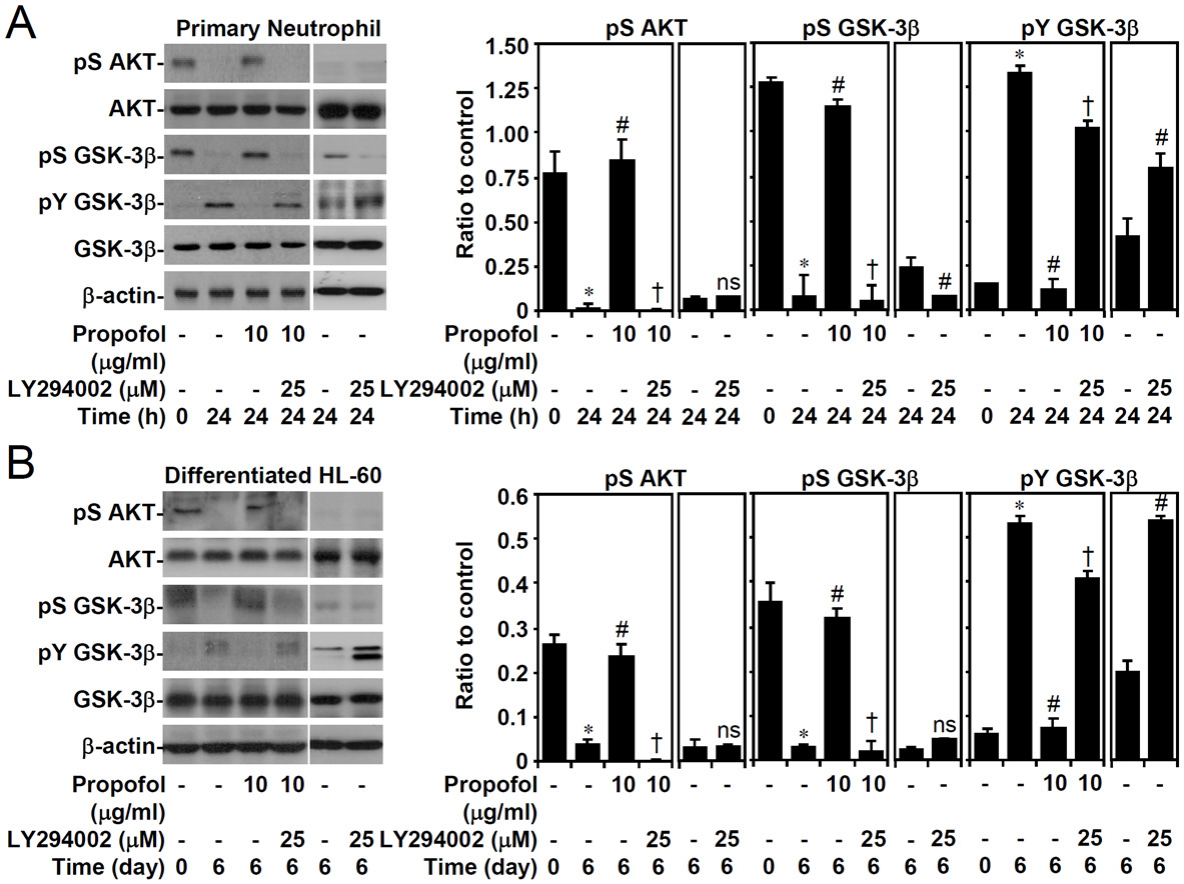
PI3-kinase-dependent propofol treatment abolishes AKT inactivation and GSK-3β activation in primary neutrophils and ATRA-differentiated granulocytic HL60 cells. (A) Isolated human primary neutrophils and (B) ATRA (1 μM)-differentiated granulocytic HL60 cells were treated with propofol with or without LY294002 treatment for the indicated time points. Western blot analysis showing the expression of phospho-AKT (Ser^473^), AKT, phospho-GSK-3β (Ser^9^), phospho-GSK-3β (Tyr^216^), and GSK3-β; β-actin was used as the loading control. One representative dataset obtained from three individual experiments is shown. The changes in the ratio of the phosphorylated protein and its total proteins are also shown. The data are shown as the means ± SD of three individual experiments. *P < 0.05 compared with untreated at 0 h. #*P <* 0.05 compared with untreated at 24 h or day 6. †*P <* 0.05 compared with propofol.

Because the activation of GSK-3β is required for neutrophils undergoing apoptosis [11], we next examined whether propofol inhibits GSK-3β activation by altering PI3-kinase/AKT signaling [7,8]. Western blot analysis revealed AKT dephosphorylation (Ser^473^), followed by GSK-3β dephosphorylation (Ser^9^, an inactive form of GSK-3β) and phosphorylation (Tyr^216^, an active form of GSK-3β) in 24 h post-culture while propofol significantly reduced such the effects in primary neutrophils (Fig 3A) and granulocyte-differentiated HL60 cells (Fig 3B). Importantly, PI3-kinase inhibitor LY294002 significantly reversed these effects induced by propofol. These findings demonstrate that propofol induces PI3-kinase/AKT-mediated GSK-3β inactivation.

### PI3-kinase signaling is required for propofol-inhibited constitutive apoptosis in isolated primary neutrophils and granulocyte-differentiated HL60 cells

**Fig 4:**
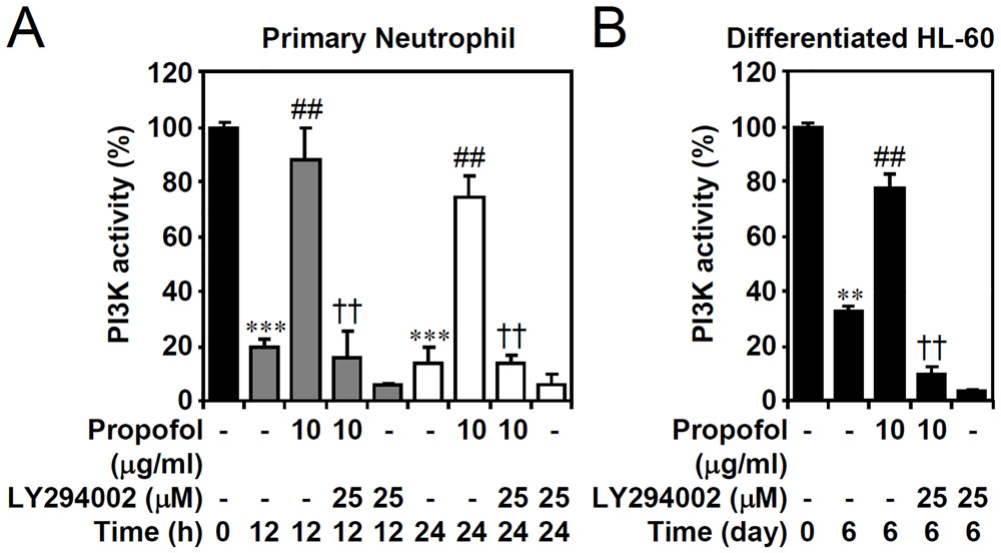
Propofol treatment reverses PI3-kinase down-regulation in primary neutrophils and ATRA-differentiated granulocytic HL60 cells. (A) Isolated human primary neutrophils and (B) ATRA (1 μM)-differentiated granulocytic HL60 cells were treated with propofol with or without LY294002 treatment for the indicated time points. PIP3 precipitate assay showing the PI3-kinase activity. The data are shown as changes with respect to the normalized values of the control. The data are shown as the means ± SD of three individual experiments. **P < 0.01 and ***P < 0.001 compared with untreated at 0 h. ##*P <* 0.01 compared with untreated at each time points. ††*P <* 0.01 compared with propofol.

**Fig 5:**
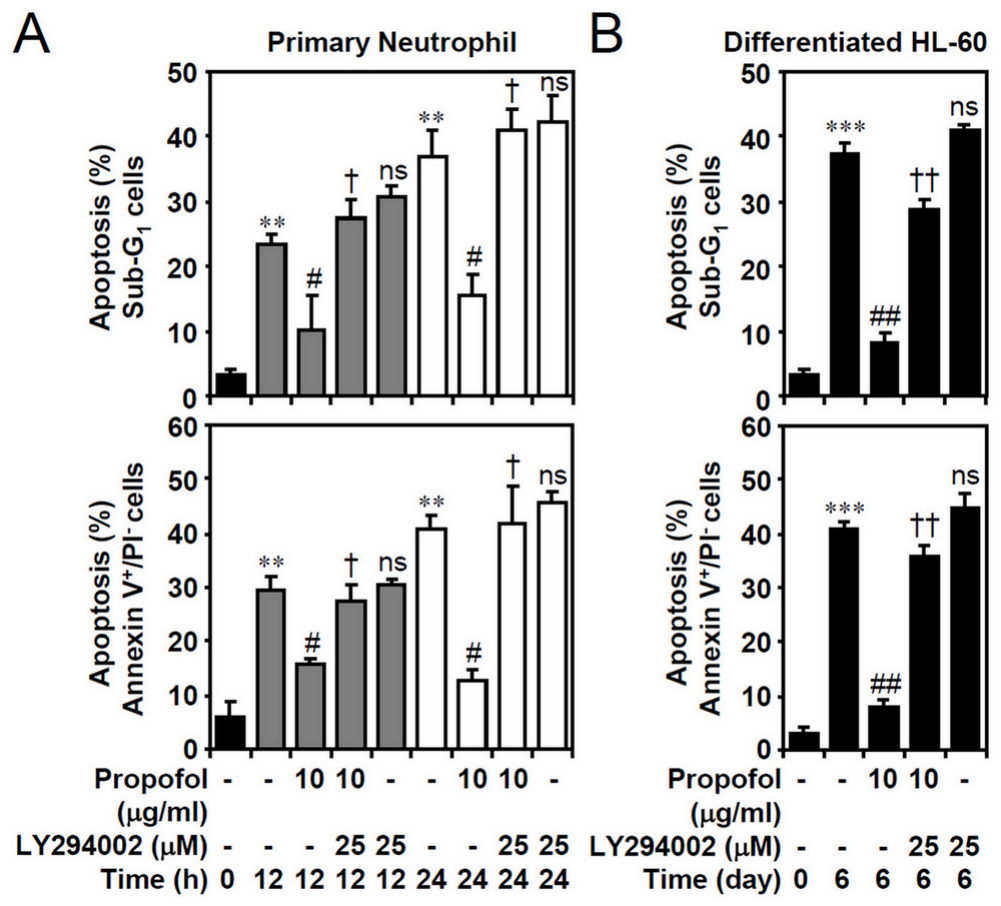
PI3-kinase-dependent propofol treatment abolishes cell apoptosis in primary neutrophils and ATRA-differentiated granulocytic HL60 cells. (A) Isolated human primary neutrophils and (B) ATRA (1 μM)-differentiated granulocytic HL60 cells were treated with propofol with or without LY294002 treatment for the indicated time points. PI and PI/Annexin V-FITC staining followed by flow cytometric analysis determined the apoptosis levels. The percentages of apoptotic cells (sub-G_1_ and Annexin V^+^/PI^-^) are shown as the means ± SD of three individual experiments. **P < 0.01 and ***P < 0.001 compared with untreated at 0 h. #*P <* 0.05 and ##*P <* 0.01 compared with untreated at each time points. †*P <* 0.05 and ††*P <* 0.01 compared with propofol. ns, not significant compared with propofol plus LY294002.

To verify the effects of propofol on PI3-kinase activity, the generation of PIP3 confirmed the inhibitory effect of propofol on PI3-kinase inactivation in primary neutrophils (Fig 4A) and granulocyte-differentiated HL60 cells (Fig 4B). These results indicate that propofol may retard PI3-kinase down-regulation in neutrophils undergoing apoptosis. We next hypothesized that a pro-survival role of propofol prevents neutrophil apoptosis by sustaining PI3-kinase activity. Nuclear PI staining followed by flow cytometric analysis showed that the primary neutrophils and granuloctyte-differentiated HL60 cells underwent apoptosis (as shown in the sub-G_1_ phase), whereas propofol significantly prevented the apoptotic effects in a PI3-kinase-mediated manner (Fig 5A and 5B, top). PI/Annexin V-FITC staining confirmed the effects of propofol on preventing early apoptosis (Fig 5A and 5B, bottom). These results demonstrate the important role of propofol in maintaining PI3-kinase activity.

### Propofol treatment, in a PI3-kinase-dependent manner, overturns Mcl-1 down-regulation, MTP loss, and caspase-3 activation in primary neutrophils and granulocyte-differentiated HL60 cells

**Fig 6:**
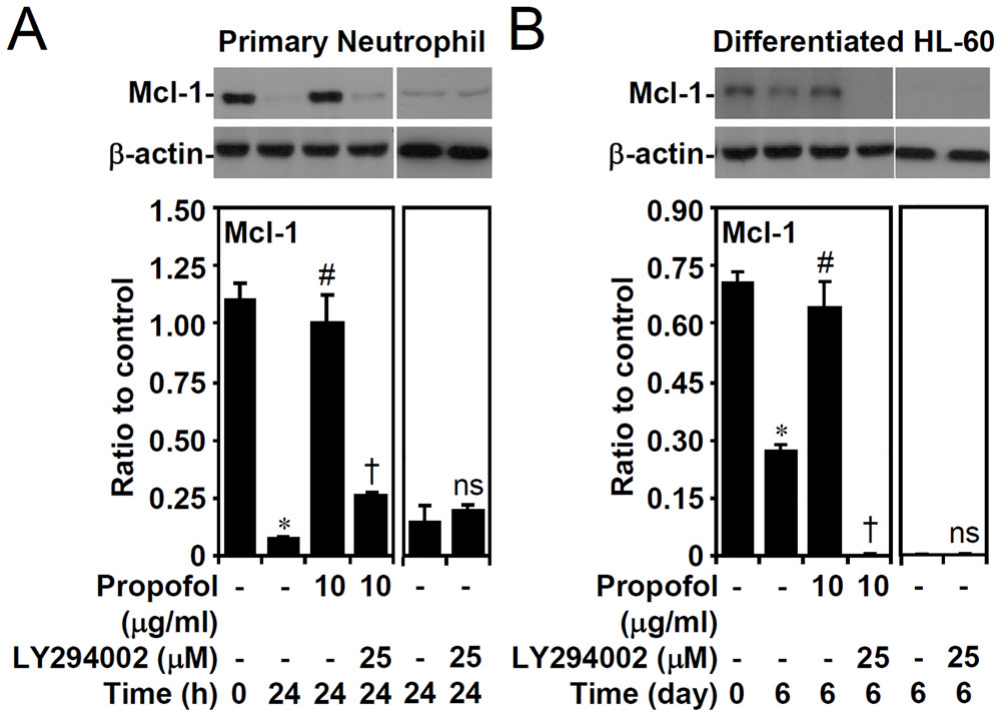
PI3-kinase-dependent propofol treatment abolishes Mcl-1 down-regulation in primary neutrophils and ATRA-differentiated granulocytic HL60 cells. (A) Isolated human primary neutrophils and (B) ATRA (1 μM)-differentiated granulocytic HL60 cells were treated with propofol with or without LY294002 treatment for the indicated time points. Western blot analysis showing the expression of Mcl-1; β-actin was used as the loading control. One representative dataset obtained from three individual experiments is shown. The changes in the ratio of Mcl-1 and β-actin are also shown. The data are shown as the means ± SD of three individual experiments. *P < 0.05 compared with untreated at 0 h. #*P <* 0.05 compared with untreated at 24 h or day 6. †*P <* 0.05 compared with propofol. ns, not significant compared with untreated at 24 h or day 6.

GSK-3β activation causes Mcl-1 downregulation, facilitating neutrophil apoptosis, as demonstrated in our previous studies. We next investigated the effects of propofol on the expression of anti-apoptotic Mcl-1. Western blot analysis showed that propofol treatment effectively reversed the destabilization of Mcl-1 in primary neutrophils (Fig 6A) and granulocyte-differentiated HL60 cells (Fig 6B). Furthermore, pharmacologically inhibiting PI3-kinase reversed the effects of propofol. These results indicate that the PI3-kinase/AKT/GSK-3β-regulated Mcl-1 determines the constitutive apoptosis of neutrophils.

**Fig 7:**
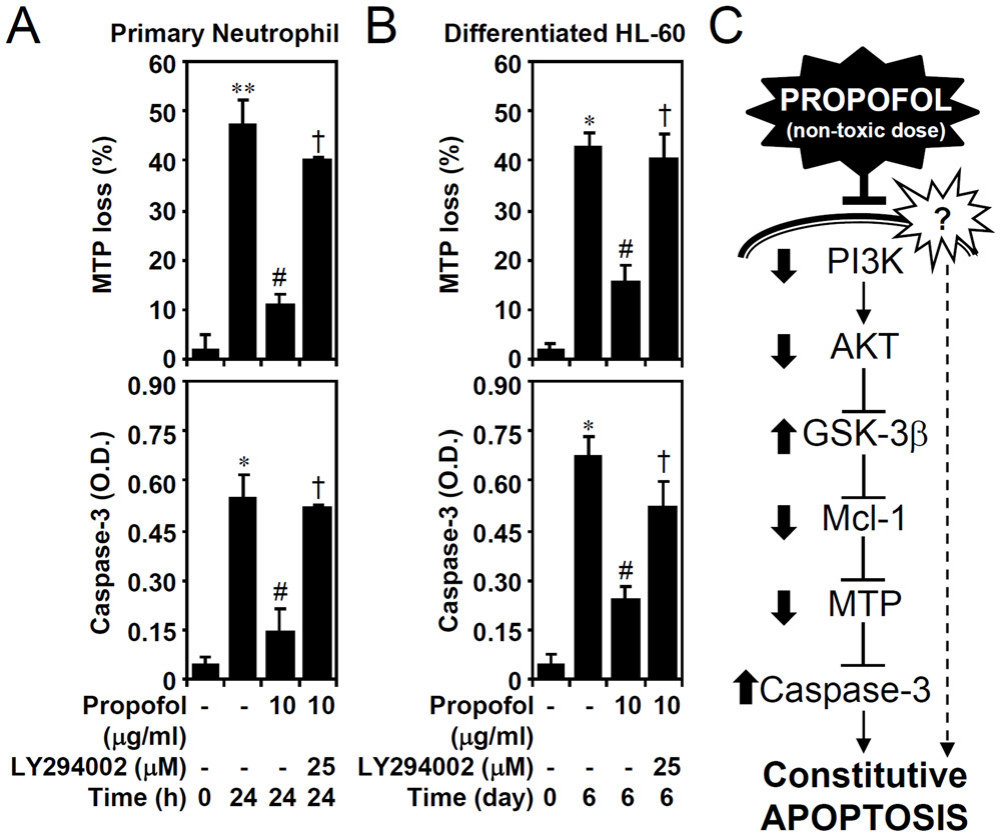
PI3-kinase-dependent propofol treatment reduces MTP loss and caspase-3 activation in primary neutrophils and ATRA-differentiated granulocytic HL60 cells. (A) Isolated human primary neutrophils and (B) ATRA (1 μM)-differentiated granulocytic HL60 cells were treated with propofol with or without LY294002 treatment for the indicated time points. A rhodamine 123-based flow cytometric analysis and caspase-3 activity assay were used to detect MTP loss (% of positive cells) and caspase-3 (OD) activation, respectively. The data are shown as the means ± SD of three individual experiments. *P < 0.05 and **P < 0.01 compared with untreated at 0 h. #*P <* 0.05 compared with untreated at 24 h or day 6. †*P <* 0.05 compared with propofol. (C) A hypothetic signaling model of propofol-mediated cytoprotection in the constitutive apoptosis of neutrophils. Through an unknown mechanism, treatment of non-toxic dose of propofol may reverse downregulation of PI3-kinase, AKT, Mcl-1, and MTP, and activation of GSK-3β and caspase-3 in neutrophils under constitutive apoptosis.

Dysregulation of the mitochondria and activation of the caspase cascade are generally involved in neutrophil apoptosis. We used lipophilic cationic fluorochrome rhodamine 123 staining followed by flow cytometric analysis and a caspase-3 activity assay kit to study the involvement of the mitochondrial pathway of apoptosis. We found that propofol significantly inhibited MTP loss and caspase-3 activation in primary neutrophils (Fig 7A) and granulocyte-differentiated HL60 cells (Fig 7B). Notably, the anti-apoptotic effects of propofol were carried out through PI3-kinase signaling. These results indicate that PI3-kinase-dependent propofol effects prevent mitochondrial damage and constitutive apoptosis in neutrophils.

## Discussion

During the progression of constitutive apoptosis in primary neutrophils and granulocyte-differentiated HL60 cells under an *in vitro* culture system, we previously demonstrated that AKT was deactivated and followed by GSK-3β activation, Mcl-1 degradation, MTP loss, caspase-3 activation, and cell apoptosis [11,12]. In this study, we provide evidence demonstrating the cytoprotective role of propofol against the apoptotic signaling pathway in both primary neutrophils and granulocyte-differentiated HL60 cells (Fig 7C). This is consistent with the cytoprotective granulocyte-macrophage colony-stimulating factors that prevent constitutive neutrophil apoptosis by maintaining PI3-kinase activity [17]; however, propofol treatment maintains PI3-kinase activity through an unknown mechanism. The significance of this study is that it is the first report to illustrate the benefits of propofol treatment on neutrophil survival. It was reported in previous studies that the induction of neutropenia is harmful to patients with sepsis [15,16] and propofol treatment confers anti-inflammatory and antioxidant effects that are beneficial for patients with sepsis and systemic inflammatory response syndrome [3]. The results of this study may also validate the cytoprotective propofol in sustaining neutrophil survival. Additionally, propofol sedation may increase the neutrophil count in the peripheral blood, so called neutrophilia [6]. The effects of propofol in an *in vivo* model of neutropenia and neutrophilia are needed to confirm the findings in this study.

Propofol treatment has dual effects on cell apoptosis and survival in phagocytes. We and others have demonstrated that a high dose (25 μg/ml or 140 μM) of propofol causes apoptosis in murine RAW264.7 macrophages [18,19]; however, the therapeutic concentration (30 μM) of propofol protects RAW264.7 cells from nitric oxide-induced apoptosis[20]. The plasma levels of propofol range between 10 and 50 μM [21]. In this study, unlike macrophages, both primary neutrophils and granulocyte-differentiated HL60 cells are susceptible to a propofol (10 μg/ml or 56 μM)-induced cytoprotective response in the process of constitutive apoptosis. The cytotoxic and survival-promoting effects of propofol are dependent on the dosage and cell type.

Constitutive apoptosis, also called spontaneous apoptosis, is essential for controlling the life-span of neutrophils after differentiation from bone marrow [1,2]. In combination with the previous findings [11–14] and the results of this work that death receptors, ROS, kinases, phosphatases, and a Bcl-2/Mcl-1/Bax imbalance activate the mitochondrial pathway of apoptosis in neutrophils, we demonstrate that propofol may confer inhibitory effects on blocking the pro-apoptotic signaling pathways. Indeed, in apoptotic neutrophils and granulocyte-differentiated HL60 cells, PI3-kinase and AKT were deactivated and followed by GSK-3β activation, Mcl-1 degradation, MTP loss, and caspase-3 activation. The inactivation of PI3-kinase may be the initial step of cellular injury.

It has been identified that aberrant ROS production may promote spontaneous neutrophil apoptosis [11,17]. ROS may affect PI3-kinase activity through an unknown mechanism. Interestingly, propofol facilitates PI3-kinase/AKT activation, protecting cells from hydrogen peroxide- and ischemia-reperfusion-induced cell injury [22,23]. Considering propofol partially maintains PI3-kinase/AKT pathway activity, it is notable that propofol may confer the antioxidant property of down-regulating oxidative stress in apoptotic neutrophils. Although propofol treatment is unable to inhibit phorbol-12-myristate-13-acetate-induced ROS generation in neutrophils [24,25]; however, during the progression of the neutrophil respiratory burst in response to formyl peptides, propofol can inhibit superoxide generation, elastase release, and chemotaxis[26,27]. Therefore, the direct or indirect molecules that propofol targets for anti-oxidation are unknown while Yang and colleagues demonstrate that propofol inhibits ROS production by blocking formyl peptide receptor [27]. In this study, we found that propofol reduces constitutive apoptosis of neutrophils and the anti-oxidative role of propofol for regulating neutrophil survival and activation needs further investigation regarding the various roles of neutrophils involved in the different pathogenesis of diseases. It is well known that propofol exerts its specific pharmacologic functions through activating the neurotransmitter gamma-aminobutyric acid (GABA) receptors [28]. However, neither the GABA_A_ nor GABA_B_ receptor mediates the inhibitory effects of propofol on ROS generation in formyl peptide-activated neutrophils [27]. The possibility that propofol has anti-oxidant properties requires further investigation even as propofol effectively rescues the deactivation of PI3-kinase in neutrophil constitutive apoptosis. Additionally, it is interesting to verify whether propofol maintains PI3-kinase through GABA receptors, while the signaling of the GABA receptor also causes PI3-kinase/AKT activation [29].

In conclusion, treatment with a therapeutic concentration (10 μg/ml or 56 μM) of propofol provided evidence verifying the molecular mechanism of propofol-induced cytoprotection against GSK-3β activation, Mcl-1 down-regulation, MTP loss, and caspase-3 activation by maintaining PI3-kinase/AKT activation in both primary neutrophils and granulocyte-differentiated HL60 cells. Prolonging the life-span of neutrophils may contribute to maintaining the antimicrobial activity and blood count of neutrophils.

## References

[1] Hofman P. Molecular regulation of neutrophil apoptosis and potential targets for therapeutic strategy against the inflammatory process. Curr Drug Targets Inflamm Allergy 2004; 3: 1–9. 15032637 10.2174/1568010043483935

[2] Fox S, Leitch AE, Duffin R, Haslett C, Rossi AG. Neutrophil apoptosis: relevance to the innate immune response and inflammatory disease. J Innate Immun 2010; 2: 216–227. 10.1159/000284367 20375550 PMC2956014

[3] Marik PE. Propofol: an immunomodulating agent. Pharmacotherapy 2005; 25: 28S–33S. 15899746 10.1592/phco.2005.25.5_part_2.28s

[4] Vasile B, Rasulo F, Candiani A, Latronico N. The pathophysiology of propofol infusion syndrome: a simple name for a complex syndrome. Intensive Care Med 2003; 29: 1417–1425. 12904852 10.1007/s00134-003-1905-x

[5] Mackenzie N, Grant IS. Propofol for intravenous sedation. Anaesthesia 1987; 42: 3–6. 3493711 10.1111/j.1365-2044.1987.tb02936.x

[6] Kim WH, Jin HS, Ko JS, Hahm TS, Lee SM, Cho HS, et al The effect of anesthetic techniques on neutrophil-to-lymphocyte ratio after laparoscopy-assisted vaginal hysterectomy. Acta Anaesthesiol Taiwan 2011; 49: 83–87. 10.1016/j.aat.2011.08.004 21982167

[7] Jope RS, Johnson GV. The glamour and gloom of glycogen synthase kinase-3. Trends Biochem Sci 2004; 29: 95–102. 15102436 10.1016/j.tibs.2003.12.004

[8] Takahashi-Yanaga F. Activator or inhibitor? GSK-3 as a new drug target. Biochem Pharmacol 2013; 86: 191–199. 10.1016/j.bcp.2013.04.022 23643839

[9] Linseman DA, Butts BD, Precht TA, Phelps RA, Le SS, Laessig TA, et al Glycogen synthase kinase-3beta phosphorylates Bax and promotes its mitochondrial localization during neuronal apoptosis. J Neurosci 2004; 24: 9993–10002. 15525785 10.1523/JNEUROSCI.2057-04.2004 PMC6730230

[10] Maurer U, Charvet C, Wagman AS, Dejardin E, Green DR. Glycogen synthase kinase-3 regulates mitochondrial outer membrane permeabilization and apoptosis by destabilization of MCL-1. Mol Cell 2006; 21: 749–760. 16543145 10.1016/j.molcel.2006.02.009

[11] Yang TT, Chen CL, Lin WC, Lin YS, Tseng PC, Hsieh CY, et al Glycogen synthase kinase-3beta inactivation is an intracellular marker and regulator for endotoxemic neutrophilia. J Mol Med (Berl) 2013; 91: 207–217. 10.1007/s00109-012-0944-6 22903504

[12] Wang CY, Yang TT, Chen CL, Lin WC, Lin CF. Reactive oxygen species-regulated glycogen synthase kinase-3beta activation contributes to all-trans retinoic acid-induced apoptosis in granulocyte-differentiated HL60 cells. Biochem Pharmacol 2014; 88: 86–94. 10.1016/j.bcp.2013.12.021 24406248

[13] Luo HR, Loison F. Constitutive neutrophil apoptosis: mechanisms and regulation. Am J Hematol 2008; 83: 288–295. 17924549 10.1002/ajh.21078

[14] Witko-Sarsat V, Pederzoli-Ribeil M, Hirsch E, Sozzani S, Cassatella MA. Regulating neutrophil apoptosis: new players enter the game. Trends Immunol 2011; 32: 117–124. 10.1016/j.it.2011.01.001 21317039

[15] Hoesel LM, Neff TA, Neff SB, Younger JG, Olle EW, Gao H, et al Harmful and protective roles of neutrophils in sepsis. Shock 2005; 24: 40–47. 15988319 10.1097/01.shk.0000170353.80318.d5

[16] Brown KA, Brain SD, Pearson JD, Edgeworth JD, Lewis SM, Treacher DF. Neutrophils in development of multiple organ failure in sepsis. Lancet 2006; 368: 157–169. 16829300 10.1016/S0140-6736(06)69005-3

[17] Klein JB, Rane MJ, Scherzer JA, Coxon PY, Kettritz R, Mathiesen JM, et al Granulocyte-macrophage colony-stimulating factor delays neutrophil constitutive apoptosis through phosphoinositide 3-kinase and extracellular signal-regulated kinase pathways. J Immunol 2000; 164: 4286–4291. 10754327 10.4049/jimmunol.164.8.4286

[18] Hsing CH, Chen YH, Chen CL, Huang WC, Lin MC, Tseng PC, et al Anesthetic propofol causes glycogen synthase kinase-3beta-regulated lysosomal/mitochondrial apoptosis in macrophages. Anesthesiology 2012; 116: 868–881. 10.1097/ALN.0b013e31824af68a 22334036

[19] Wu KC, Yang ST, Hsu SC, Chiang JH, Hsia TC, Yang JS, et al Propofol induces DNA damage in mouse leukemic monocyte macrophage RAW264.7 cells. Oncol Rep 2013; 30: 2304–2310. 10.3892/or.2013.2722 24008596

[20] Chang H, Tsai SY, Chang Y, Chen TL, Chen RM. Therapeutic concentrations of propofol protects mouse macrophages from nitric oxide-induced cell death and apoptosis. Can J Anaesth 2002; 49: 477–480. 11983662 10.1007/BF03017924

[21] Gepts E, Camu F, Cockshott ID, Douglas EJ. Disposition of propofol administered as constant rate intravenous infusions in humans. Anesth Analg 1987; 66: 1256–1263. 3500657

[22] Wang B, Shravah J, Luo H, Raedschelders K, Chen DD, Ansley DM. Propofol protects against hydrogen peroxide-induced injury in cardiac H9c2 cells via Akt activation and Bcl-2 up-regulation. Biochem Biophys Res Commun 2009; 389: 105–111. 10.1016/j.bbrc.2009.08.097 19703415 PMC3631547

[23] Wang HY, Wang GL, Yu YH, Wang Y. The role of phosphoinositide-3-kinase/Akt pathway in propofol-induced postconditioning against focal cerebral ischemia-reperfusion injury in rats. Brain Res 2009; 1297: 177–184. 10.1016/j.brainres.2009.08.054 19703434

[24] Davidson JA, Boom SJ, Pearsall FJ, Zhang P, Ramsay G. Comparison of the effects of four i.v. anaesthetic agents on polymorphonuclear leucocyte function. Br J Anaesth 1995; 74: 315–318. 7718379 10.1093/bja/74.3.315

[25] Erol A, Reisli R, Reisli I, Kara R, Otelcioglu S. Effects of desflurane, sevoflurane and propofol on phagocytosis and respiratory burst activity of human polymorphonuclear leucocytes in bronchoalveolar lavage. Eur J Anaesthesiol 2009; 26: 150–154. 10.1097/EJA.0b013e328319bfeb 19142090

[26] Murphy PG, Ogilvy AJ, Whiteley SM. The effect of propofol on the neutrophil respiratory burst. Eur J Anaesthesiol 1996; 13: 471–473. 8889420 10.1046/j.1365-2346.1996.00003.x

[27] Yang SC, Chung PJ, Ho CM, Kuo CY, Hung MF, Huang YT, et al Propofol inhibits superoxide production, elastase release, and chemotaxis in formyl peptide-activated human neutrophils by blocking formyl peptide receptor 1. J Immunol 2013; 190: 6511–6519. 10.4049/jimmunol.1202215 23670191

[28] Trapani G, Altomare C, Liso G, Sanna E, Biggio G. Propofol in anesthesia. Mechanism of action, structure-activity relationships, and drug delivery. Curr Med Chem 2000; 7: 249–271. 10637364 10.2174/0929867003375335

[29] Xu J, Li C, Yin XH, Zhang GY. Additive neuroprotection of GABA A and GABA B receptor agonists in cerebral ischemic injury via PI-3K/Akt pathway inhibiting the ASK1-JNK cascade. Neuropharmacology 2008; 54: 1029–1040. 10.1016/j.neuropharm.2008.01.014 18410948

